# Coding of shape from shading in area V4 of the macaque monkey

**DOI:** 10.1186/1471-2202-10-140

**Published:** 2009-11-30

**Authors:** Fabrice Arcizet, Christophe Jouffrais, Pascal Girard

**Affiliations:** 1 Université de Toulouse UPS, Centre de recherche Cerveau et Cognition, Toulouse, France; 2 CNRS, CerCo, Toulouse, France; 3 Université de Toulouse UPS, IRIT, Toulouse, France; 4 IRIT, CNRS, Toulouse, France; 5 Department of Neurobiology Bisley's Lab, UCLA, Los Angeles, USA

## Abstract

**Background:**

The shading of an object provides an important cue for recognition, especially for determining its 3D shape. However, neuronal mechanisms that allow the recovery of 3D shape from shading are poorly understood. The aim of our study was to determine the neuronal basis of 3D shape from shading coding in area V4 of the awake macaque monkey.

**Results:**

We recorded the responses of V4 cells to stimuli presented parafoveally while the monkeys fixated a central spot. We used a set of stimuli made of 8 different 3D shapes illuminated from 4 directions (from above, the left, the right and below) and different 2D controls for each stimulus. The results show that V4 neurons present a broad selectivity to 3D shape and illumination direction, but without a preference for a unique illumination direction. However, 3D shape and illumination direction selectivities are correlated suggesting that V4 neurons can use the direction of illumination present in complex patterns of shading present on the surface of objects. In addition, a vast majority of V4 neurons (78%) have statistically different responses to the 3D and 2D versions of the stimuli, while responses to 3D are not systematically stronger than those to 2D controls. However, a hierarchical cluster analysis showed that the different classes of stimuli (3D, 2D controls) are clustered in the V4 cells response space suggesting a coding of 3D stimuli based on the population response. The different illumination directions also tend to be clustered in this space.

**Conclusion:**

Together, these results show that area V4 participates, at the population level, in the coding of complex shape from the shading patterns coming from the illumination of the surface of corrugated objects. Hence V4 provides important information for one of the steps of cortical processing of the 3D aspect of objects in natural light environment.

## Background

A fundamental issue of visual perception is to understand how the brain represents the 3D shape of an object from the 2D patterns that project onto the retina [1]. While it is clear that stereopsis and motion parallax are potent sources of 3D information, human observers routinely extract 3D shape from static monocular cues and flawlessly recognize 2D images or drawings in which these features are the only ones available. To achieve this, humans rely upon many factors such as texture gradients, the presence of particular junctions and edges, or the pattern of shadows. In natural situations, variations of illumination direction produce large variations of shading patterns that complicate the recognition of a 3D object. Several studies have revealed deficits in recognizing faces or objects under various shadow conditions [2,3], and in estimating surface curvature based on shading [4-6] or perceptual ambiguities [7]. If one introduces a display change in matching experiments [8], the recognition of objects, with the exception of human faces, does not appear to depend on the direction of illumination [9,10]. So, humans are able to recognize shapes within a highly variable environment and are able to use 2D pictorial cues, like shading, to form vivid 3D percepts [1,11,12]. The question then arises: What neuronal mechanisms underlie such a process of shape recognition?

The precise mechanisms by which the brain extracts the different sources of monocular 3D information and combines them to identify an object remain unknown. In particular, few studies have investigated the question of 3D shape from shading [13]. fMRI studies on humans indicate a participation of both dorsal and ventral pathways [14-16]. More recently, the question was remarkably well approached by Georgieva et al (2008) [13], who used more controls and avoided other 3D cues (edges, vertices) than shading alone. The results of this study underlined the importance of the caudal inferotemporal gyrus and ruled out the intraparietal sulcus as a site for the extraction of 3D shape from shading.

Single-unit studies have suggested that V4 neurons play an important role in shape from shading. For instance, Hanazawa and colleagues [17,18] showed that V4 neurons are selective to shading orientation with a vertical bias. Furthermore, curvature was critically represented in V4 [19-21] in the form of 'volumetric primitives'. Hence, the representation of curvature in V4 might reflect a necessary processing step of shape from shading before achieving invariance to shading variations that occur in higher level regions [22]. The main psychophysical counterpart is that shading is particularly important for the analysis of curved surfaces and, according to Todd [1], perceptual constancy of objects can be achieved through a curvature-based representation of shapes. We thought it was important to examine further the selectivity of V4 neurons to shapes defined by shadings. We expect single unit studies, which stand at a different level of analysis, to potentially reveal shape from shading-related mechanisms in V4. Finally and importantly, it should be stressed that macaque monkeys are a valuable model for the study of 3D shape from shading at the single cell level as it has been demonstrated that they can perceive depth from shading cues in behavioral tasks [23].

The aim of our study was to explore the encoding of 3D shape from shading in area V4 of the awake macaque monkey. The particularity of shape from shading implies that shape and illumination are intimately intertwined to create a 3D percept. A light source illuminating the surface of an object containing irregularities such as hollows and bumps, inescapably creates a pattern of dark and light regions that is specific to the shape of the object. If other cues are unavailable, the brain needs to use this pattern of shading to infer the 3D aspect of the surface. We first aimed to test if V4 cells are selective to 3D shapes defined by illumination that creates different patterns of shading. In order to assess this, we used a set of 8 different naturalistic 3D shapes illuminated from 4 directions (from below, the left, the right and above). Because the pattern of shading varies markedly when the direction of illumination varies, we computed several indices to check if the V4 cells responded invariantly to the same 3D shape illuminated from different directions or if their selectivity was biased towards vertical illumination directions. Finally, we tested the selectivity to 3D shape from shading per se by using 3 different types of 2D controls. These controls share low-level parameters with the 3D stimuli and by changing the spatial organization of the shading patterns, they loose their 3D aspect.

Our results show that most individual V4 neurons do not show a strong selectivity to individual 3D shapes defined by shading. We also noticed a weak selectivity to illumination directions with no preference for vertical axes. Furthermore V4 neurons do not prefer systematically the 3D version of the stimuli with respect to the 2D controls. However, 3D stimuli and 2D controls could be clearly separated by a cluster analysis of V4 single cell responses, suggesting that shape from shading is a cue encoded at the population level.

## Methods

### Animals and setup

Two adult rhesus monkeys, one female (monkey T) and one male (monkey Z), weighing 3 and 6 kg respectively, were implanted with head fixation devices (Crist Instruments, Hagerstown, MD). Surgical operations were performed under general anesthesia and sterile conditions. Anesthesia was induced by ketamine (16 mg/kg IM). Maintenance of anesthesia was achieved with a mixture of alphadolone/alphaxolone (Saffan, 15 mg/kg/h IV or slightly more if required). A pain reliever, ketoprofen (Ketofen, 1 mg/kg IM) and systemic antibiotics (extencilline 600000 UI IM) were administrated at the beginning of the surgery.

Once monkeys were trained to perform a simple visual fixation task, we performed a second surgery to implant a recording chamber over a 2 cm diameter craniotomy. The surgery was performed under the same conditions, except for an additional injection of methylprednisolone (solumedrol, 1 mg/kg IM) to prevent brain edema. Although we cleaned within the chamber daily, guide tubes were required because we did not scrape the thickening dura. Animals were sacrificed by an overdose of pentobarbital and fluorescent dyes were injected to localize the recording sites and confirmed the location of recordings in V4. Histological analyses on both monkeys confirmed that we recorded cells in the anterior part of dorsal V4. An anatomical description of the region of recordings can be found in Arcizet et al. 2008 [24]. All animal procedures complied with guidelines of the European Ethics committee on Use and Care of Animals.

To perform the task, the animals were seated in a primate chair, with their head restrained. An ISCAN infrared eye-tracking system (120 Hz) monitored eye positions by tracking the corneal reflection of a focused infrared LED through a CCTV camera with a 250-mm lens. The experiments were run using CORTEX software (courtesy of NIMH), which controlled stimulus presentation and data acquisition. Tungsten-in-glass microelectrodes (Thomas Recording, Germany) were used to record extracellular neuronal activity. Action potentials from single units were sorted online (MSD, AlphaOmega, Israel).

### Stimuli and protocol

Stimuli consisted of pictures of randomly deformed spheres similar to those used in studies [25] and [13]. The illumination falling on concavities and convexities of the spheres produced patterns of shading that made the stimuli look like vivid pictures of realistic 3D objects. We used 8 different distorted spheres (termed 3D shapes). These stimuli were illuminated with a Lambertian light source (with no specular component) coming from 4 different directions (below, right side, left side or above).

**Figure 1:**
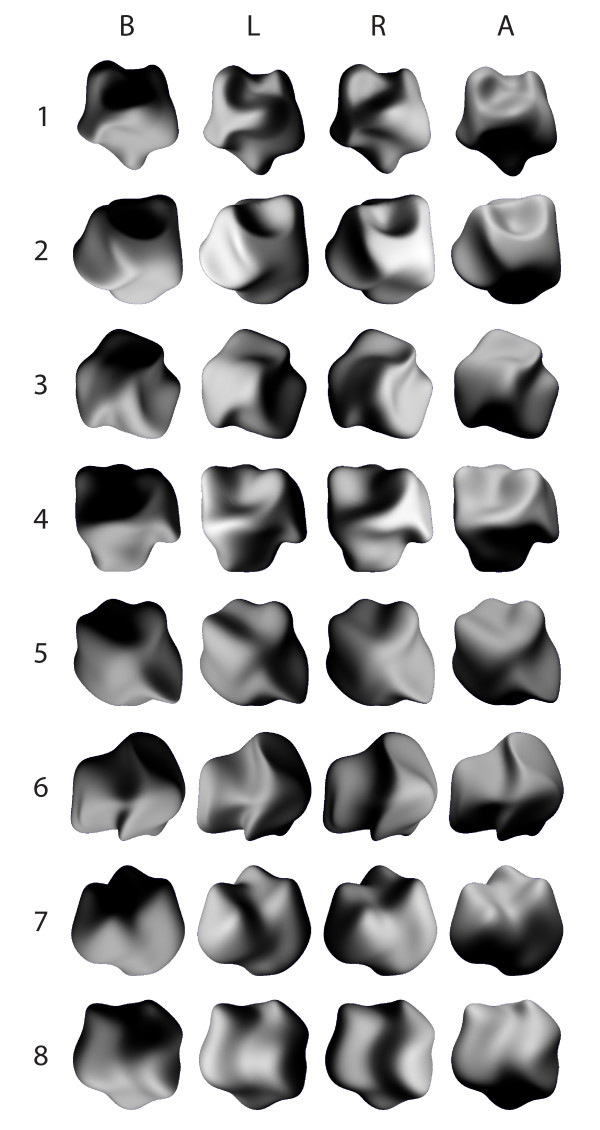
**Illustration of the 32 stimuli**. The eight 3D shapes (rows) rendered with the four light source directions (columns). The illumination used for rendering is mentioned at the top of the figure (below (B), left (L), right (R) and above (A)).

**Figure 2:**
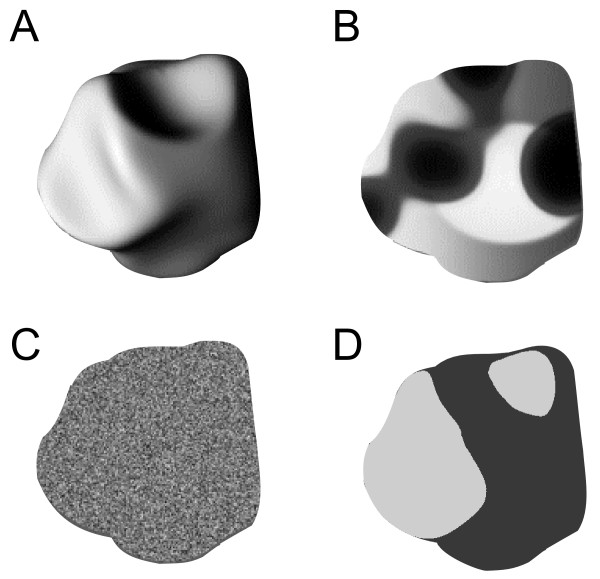
**Illustration of one 3D shape and its three 2D control stimuli**. (A) Original 3D: shape #2 illuminated from the left (#2L). (B) Blob control: 2D percept created by redistribution of sections from the original image within the outline. (C) Random control: random distribution of the original image pixels. (D) Posterized control with 2 grey levels. The Random and Posterized stimuli were used in different subsets of neurons.

Therefore, the set of original stimuli consisted of 32 images of 3D shapes (Figure 1). As discussed in [13], it is not possible to design 2D controls of these stimuli that look flat and keep all low-level parameters. Hence, several types of 2D controls were necessary (Figure 2): (1) The "Blob" control consisted of a texture made of random distribution of 'blobs' taken within the outline of the original 3D stimulus (Fig. 2B). Blobs and random stimuli were designed by the group of Rufin Vogels (University of Louvain) with 3D studio max software. (2) The "Random" control consisted of a random distribution of the pixels of the original 3D image (Fig. 2C). (3) The "Posterized" control: each image of 3D shape was posterized (continuous gradation of tones replaced with fewer levels using Paint shop pro software) with two gray levels (Fig. 2D). Posterized stimuli roughly correspond to the spatial pattern of black and white patches contained in the original stimuli. These stimuli are identical to those called 'unshaded blobs' in [13]. None of the Blob and Random controls led to the perception of vivid 3D shapes. Posterized stimuli with 2 grey levels gave no 3D percept, although those with 4, 6 and 8 levels did (data in human subjects, not shown).

**Figure 3:**
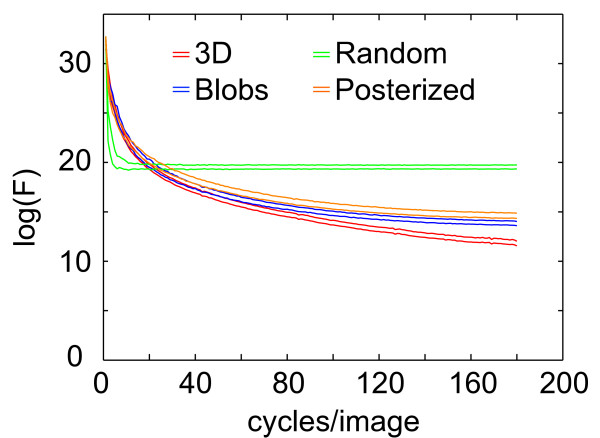
**Power spectra (F) of the different classes of stimuli**. Power spectra are averaged over the 4 or 8 different shapes and collapsed over orientations. The distance between 2 curves with the same color represent the standard deviation.

The mean luminance of the 3D images did not differ by more than 0.9% from that of their respective Blob and Posterized controls. As expected, the difference was less than 0.02% with the Random controls. Figure 3 shows that the mean power spectrum collapsed over all orientations was similar for 3D, Blob and Posterized stimuli (but not for Random stimuli).

We had a total of 96 different stimuli (8 outlines * 4 directions of illumination * 3 contents [3D shapes, Blob, (Random or Posterized)]). The stimuli were gamma corrected on a 21" CRT monitor (Iiyama vision master pro512) placed at 57 cm from the eyes of the monkeys. We adapted stimulus size to eccentricity rather than precisely matching stimuli to measured receptive field (RF). Practically, during the recording sessions, stimuli were chosen among 4 identical but scaled sets of 2, 3, 4 or 5 degrees of visual angle and presented at the center of the receptive field.

The monkeys were first trained to maintain fixation within a 2-degree square window. The monkey had to keep fixating a 0.1 degree gray central spot for a variable delay (400 to 600 ms) before the stimulus was flashed for 250 ms. After the stimulus was turned off, the fixation spot remained on for a variable delay (350 to 400 ms). Only trials completed without breaking fixation were rewarded with a drop of water and kept for off-line analysis

For each isolated neuron, we first roughly mapped the receptive field with dark, light or colored hand-moved bars. In order to quickly find the RF center, we recorded the neuronal responses to small squares (dark or light) flashed for 25 ms at 36 positions selected pseudo-randomly in a square grid. RF sizes and eccentricities were in agreement with previous studies [26]. Once the RF mapping was achieved, we recorded 5 to 10 trials for each stimulus. Stimuli were presented in pseudo-random order.

### Data analysis

We defined two 250 ms epochs, one corresponding to the baseline and the other to response activities of the neurons. The baseline epoch began during the initial fixation period, 400 ms before stimulus onset. The response epoch began 50 ms after stimulus onset. Mean response rates (spikes/s) were computed for both epochs. Baseline rates were generally low (average +/- SD: 6.2 +/- 0.6 spikes/s). Data analysis on response rates with or without subtraction of the baseline activity yielded similar results. Thus, results reported in the paper are from the recorded response rates, without subtraction of the baseline activity.

All 8 3D shapes have a marked different aspect because of the presence of surface concavities or convexities. V4 neurons are expected to be selective to the smooth curves enhanced by shading since previous studies have demonstrated that they are sensitive to contour elements [19,21]. An interesting question is the influence of illumination direction on 3D shape selectivity. To assess neuronal selectivity to 3D shape and illumination direction, we computed a two-way non-repeated factorial ANOVA with 3D *shape *and *illumination direction *(ID) as independent factors. The threshold of significance was fixed at 5%.

We also computed a three-way non-repeated factorial ANOVA (with *shape, content*, and *illumination direction *as independent factors) to determine the selectivity of cells to content, and a post-hoc comparison (Tukey test) to compare the mean responses to different types of stimuli content (3D, Blobs and Random/Posterized). In order to quantify the magnitude of the selectivity for 3D shape and illumination direction, we computed two modulation indices inspired by Ref. [27]. For each neuron, we determined the combination of 3D shape and illumination direction that induced the maximal response. We defined the 3D shape selectivity index (SSI) as [Rmax - Rmin]/[Rmax +Rmin], where Rmin and Rmax are the minimal and maximal response among the 3D shapes, respectively. The Illumination Selectivity Index (ISI) was defined similarly. A value of 0 indicates absence of modulation by the factor (3D shape or illumination direction), whereas a value of 1 indicates a strong selectivity. Since an ANOVA does not quantify the strength of selectivity, we also computed a ω^2 ^association index derived from the two-way ANOVA to assess the tuning to both factors (3D shape and illumination direction). In contrast to selectivity indices (SSI and ISI), ω^2 ^captures both the mean and the trial-to-trial variability instead of using the responses to the least or the best effective stimulus [28]. This ANOVA ω^2 ^index is defined as:

where *SS *is the sum of squares, *MS *the mean squares and *df *the degree of freedom. This index ranges between 0 and 1; a value of 1 indicates a strong selectivity whereas a value of 0 indicates no selectivity. Neurons were considered to be highly selective to 3D stimuli or to illumination direction when ω^2 ^was above a threshold of 0.10 [28].

#### Ranking

In addition, we performed a ranking analysis [29] to test how the controls affect the tuning to 3D shapes and a cluster analysis to evaluate to what extent 3D stimuli could be segregated from controls by the V4 population. We performed this analysis to assess the preservation of selectivity to the 3D shape stimuli across modification of the content. For each neuron, responses to each 3D stimulus were normalized and ranked in descending order (the best 3D stimulus had the rank of 1). Then, for the same neuron, the obtained rank was used as a reference to rank the responses to the different corresponding types of control stimuli (Blobs, Random and Posterized). The procedure was repeated for each neuron and then, for each class of stimuli, we averaged the responses for each rank across all neurons. Since the reference ranking comes from the 3D stimuli, a flat ranking curve for a given control class would mean that the cell population preference for that control and the 3D shapes is markedly different. Conversely, a superimposed or parallel curve means that the shape preference is preserved across stimulus classes.

#### Cluster analysis

Finally, we used a hierarchical cluster analysis to obtain a visual representation of the neuronal responses at the population level. The purpose of cluster analysis is to gather the stimuli into successively larger clusters, using a measure of distance between neuronal responses. Results are illustrated with a hierarchical tree or dendrogram. We used the Ward's linkage method on Euclidean distances obtained from standardized responses (Statistica software) to perform the analysis. This method uses an analysis of variance approach to evaluate the distances between clusters. Hence it seeks to choose the successive clustering steps so as to minimize the increase in the error sum of squares found at each level (see [30] for details concerning this method).

## Results

We recorded 124 V4 neurons in the right hemispheres of two monkeys (Monkey T, 93; Monkey Z, 31). A vast majority of neurons (119/124 or 96%) showed a mean firing rate that increased significantly during stimulus presentation (T-test, p < 0.05). All the cells were tested with 3D shapes and Blobs, but among the 119 responsive cells (Monkey T, 90; Monkey Z, 29), only 46 cells were tested with the Random controls, and 73 cells with the Posterized controls (all cells in this last sample were tested with only four of the eight shapes). We used the population of 119 responsive cells for subsequent analysis.

**Figure 4:**
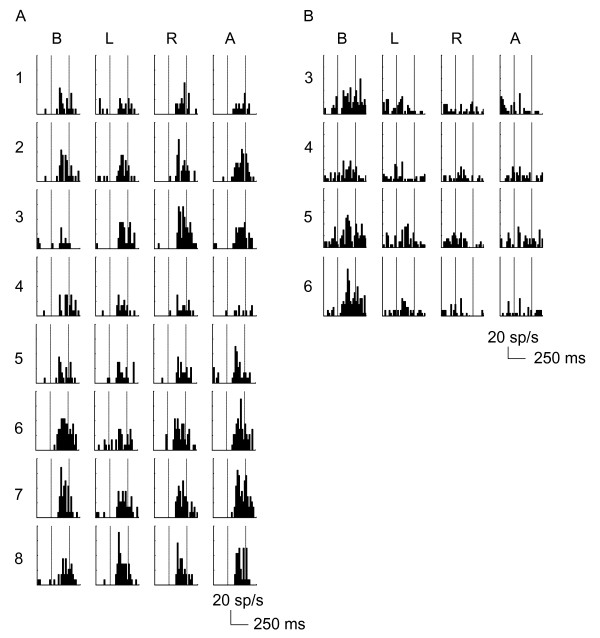
**Selectivity of 2 V4 neurons to 3D shapes illuminated from different directions**. Peristimulus time histograms from two V4 cells in response to the different combinations of 3D shapes and illumination directions. Each row corresponds to a given 3D shape and each column corresponds to a given illumination direction (Below, Left, Right and Above), respectively. Second and third vertical lines on each PSTH represent stimulus on- and offset (250 ms duration). (A) A Neuron which is highly selective for 3D shape but weakly for illumination direction (ANOVA, main effect, 3D shape; p < 0.001, illumination direction; p = 0.046). (B) A Neuron which is highly selective for illumination direction but less for 3D shape (ANOVA, main effect, 3D shape; p = 0.006, illumination direction; p < 0.001).

We computed a two-way ANOVA (*shape number *× *Illumination Direction*) on mean responses to 3D stimuli. Figure 4 shows the responses of 2 example cells to all the possible combinations of 3D shapes and illumination directions (8*4 for example A and 4*4 for example B). The neuron in Figure 4A shows a strong modulation by 3D shape and a minor effect of illumination direction. Its response is maximal for 3D shape # 7 when illuminated from above, but stays high for the 3 remaining illumination directions, yet with a lower response when illumination comes from the left side. Conversely, Figure 4B illustrates an example of neuron that is strongly modulated by illumination direction but less by 3D shapes. Both neurons show a significant effect of 3D shapes and illumination direction respectively (ANOVA, main factor, p < 0.05). Statistical analysis (ANOVA) showed that among the 119 cells, 45% (53/119) of the neurons were significantly modulated by 3D shape and 55% (65/119) of cells were significantly modulated by illumination direction. Among these neurons, 38% (45/119) showed a significant interaction between 3D shapes and illumination direction.

**Figure 5:**
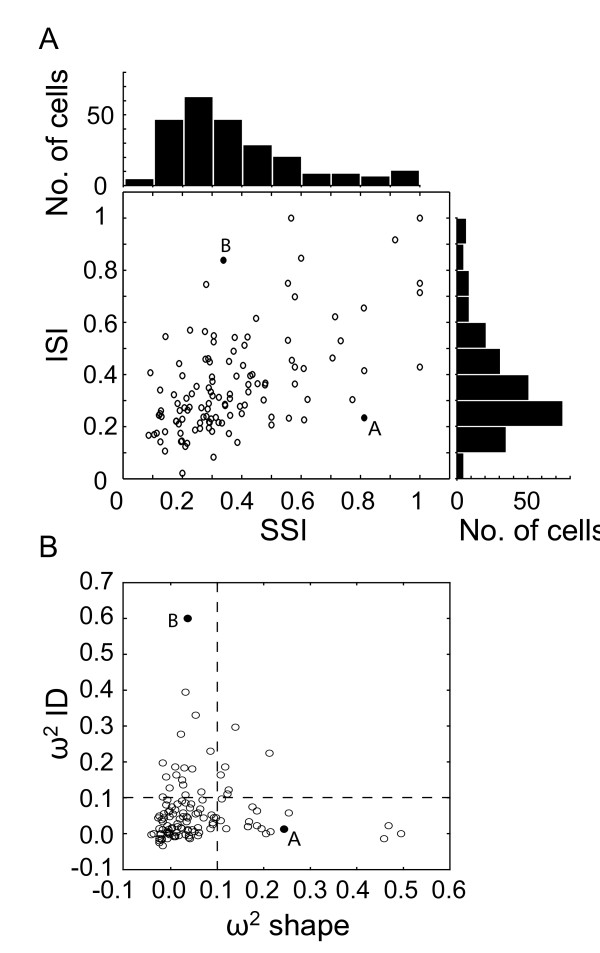
**Selectivity indices**. (A) Scatter plot of the illumination selectivity index (ISI) as a function of the shape selectivity index (SSI), with their respective distributions plotted on the top and right sides of the scatter plot. The median values of SSI and ISI distributions are 0.30 and 0.31 respectively. The black dots represent the neurons illustrated in Figures 4A (SSI = 0.81, ISI = 0.23) and 4B (SSI = 0.31, ISI = 0.83). (B) The ANOVA based ω^2 ^index for illumination direction plotted as a function of the ω^2 ^index for shape. The black dots represent the neurons illustrated in Figures 4A & B. Dashed lines represent the thresholds of 0.10 that we used to determinate that a cell is highly selective to shape or illumination direction.

In order to quantify the selectivity to both 3D shape and illumination direction, we computed two selectivity indices (3D Shape Selectivity Index, SSI; Illumination Selectivity Index, ISI; see Methods). Figure 5A shows a scatter plot and distribution histograms of the SSI and ISI computed within the population (n = 119). SSIs for neurons illustrated in Figure 4A and 4B are 0.81 and 0.31 respectively. The distribution of SSI (Figure 5A, top axis) has a median value of 0.30, indicating that the V4 population shows a weak modulation of responses according to the different 3D shapes. Similarly, the distribution of the ISI (Figure 5A, right axis) has a median value of 0.31 indicating that there is a broad tuning to illumination direction across the population. The neurons in Figure 4A and 4B have ISIs of 0.23 and 0.83 respectively. Surprisingly, the scatter plot (Figure 5A) shows that 3D shape selectivity is significantly and positively correlated with Illumination Direction (Pearson correlation; r = 0.566, p < 0.001) suggesting that the determination of the illumination direction is essential to elaborate the shape from shading selectivity. At this stage of the analysis, our results indicate that individual V4 neurons do not show a marked preference across the 3D shapes illuminated for different directions but they could use illumination direction to encode shape from shading.

A better quantitative measure of the tuning is provided by the ω^2 ^index from the ANOVA (see Methods). We computed this index for each neuron for both the shape of the stimuli (ω^2^S) and the illumination of the stimuli (ω^2^ID). Figure 5B shows the scatter plot of both indices. The median values of ω^2^S and ω^2^ID were 0.028 and 0.036 respectively, which, along with SSI and ISI indices, confirms that the tuning for both features was weak. Only 23 cells (19%) have a ω^2^S above 0.10, the threshold value above which a neuron is considered selective. Similarly, 23 cells have a ω^2^ID above 0.10 but only 6 have both indices above threshold. This is reinforced in the scatter plot of Figure 5B that shows an absence of correlation between both indices (Pearson correlation; r = 0.005, p = 0.960). However, when we focused on the pool of neurons selective to the 3D shape (according to the threshold of 0.10, right side of the dashed line perpendicular to the x-axis), we observed a weak negative correlation between tuning to 3D shape and illumination (Pearson correlation; r = -0.421, p = 0.0007). This could mean that the shape selectivity is associated with a tendency to the invariance to the direction of illumination in a few cells, like the one in Figure 4A. Furthermore, we observed no correlation between tuning to shape and direction of illumination for the pool of illumination direction selective cells (Pearson correlation; r = 0.039, p = 0.826, upper side of the dashed line parallel to the x-axis).

**Figure 6:**
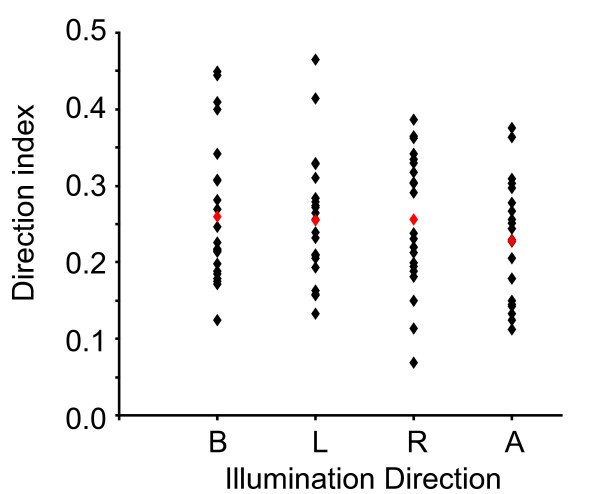
**Direction index**. Scatter plot of direction indices are plotted as a function of illumination direction for illumination direction selective cells (n = 23). One point corresponds to the index computed for one illumination direction selective cell at one illumination direction. Red dots represent the mean direction index for each illumination direction. There is no preference for a given illumination direction (ANOVA, p = 0.586).

According to Hanazawa and Komatsu [17], one could expect a stronger modulation of V4 Illumination Direction selective cells (ω^2^ID > 0.10, n = 23) when stimuli are illuminated from vertical directions (above and below). In order to examine this point, we used for each selective neuron a set of 4 indices computed from the responses to its preferred 3D shape only. For each illumination direction, we defined the Direction index as Rdirection/(Σ R 4 directions), where 'Rdirection' is the response to a given direction and 'Σ R 4 directions' is the sum of the responses to each of the four illumination conditions. As a consequence, we obtained 4 Direction indices for each neuron. Figure 6 shows the scatter plot of these indices computed from the responses of the illumination direction selective cells (n = 23). Interestingly, there is no significant response bias towards a given illumination direction, even for the 'from above' direction (ANOVA, F(3) = 0.684, p = 0.564). The same result is obtained if we extend the analysis to the whole pool of cells. This result is consistent with the weak selectivity to illumination direction represented in Figure 5.

**Figure 7:**
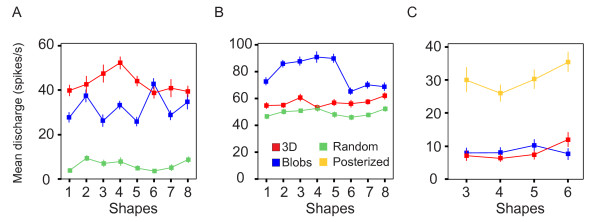
**Examples of 'content' cells responses**. Each plot shows the mean responses of a single neuron to each 3D shape (the 4 different illumination directions are pooled together). Neurons preferring 3D stimuli (A), Blobs stimuli (B) and Posterized stimuli (C) are plotted separately. Colored curves correspond to the types of stimuli (see legend). Error bars correspond to standard error of the mean.

Genuine selectivity to 3D cannot be studied without appropriate 2D controls. In this section, we explore if V4 neurons are sensitive to the 3D or 2D aspect of the content of the stimuli with the use of several control stimuli (see Methods). The results of a three-way ANOVA (*shape × content × illumination direction*) show that 93 cells (78%) responded differently according to the content (3D/Blob/[Random or Posterized], main effect, p < 0.05). We termed these 93 cells 'content' cells and restrict further response comparisons of 3D stimuli with control stimuli to this subpopulation only. Figure 7 shows three examples of 'content' cells preferring the 3D, Blob, or Posterized stimuli respectively. Among the population of 'content cells', 60 responded differently to the 3D shapes and 2D Blobs (Tukey test, p < 0.05), of which 24 neurons preferred the 3D stimuli (Figure 7A) and 36 cells gave better responses to the Blob stimuli (Figure 7B). Among the 'content' population, 40 cells were recorded with the Random control stimuli. The Tukey post-hoc comparison (p < 0.05) showed that responses to Random stimuli were mostly lower than to 3D shapes and Blobs (29/40, 72.5%). The remaining 53 'content' cells were recorded with the Posterized stimuli. We observed similar percentages of 'content' cells that responded more, equally or less to Posterized stimuli than to 3D stimuli (34%, 32% and 34% respectively, Tukey-test). Table 1 (additional file 1) shows the mean population response for each cell type determined by the ANOVA. There was no evidence of stronger responses to 3D stimuli than to their respective controls.

In the first part of the analysis, we characterized 2 subpopulations of neurons that were selective to 3D shapes or illumination direction according to their ω^2 ^indices (ω^2 ^> 0.10). In these subpopulations, a vast majority of cells are also 'content' selective (20/23 for shape selective cells and 20/23 for illumination selective cells). Following our definition of 'content' cells, the selectivity to both factors should be affected by the presentation of the 2D controls. To assess this point, we computed the selectivity indices ω^2 ^for the responses to 2D controls (Blob and Random or Posterized). A majority of cells selective to 3D shape remained selective to the shape of Blob controls (18/23, ω^2^S > 0.10) and fewer cells were also selective (10/23) to Random or Posterized controls (1 and 9 cells respectively). On the other hand, the selectivity to illumination direction is more disrupted by control stimuli as fewer cells remained selective for illumination direction in Blobs stimuli (ω^2^ID, n = 7/23) and 9 for Random and Posterized stimuli (1 and 8 cells respectively).

**Figure 8:**
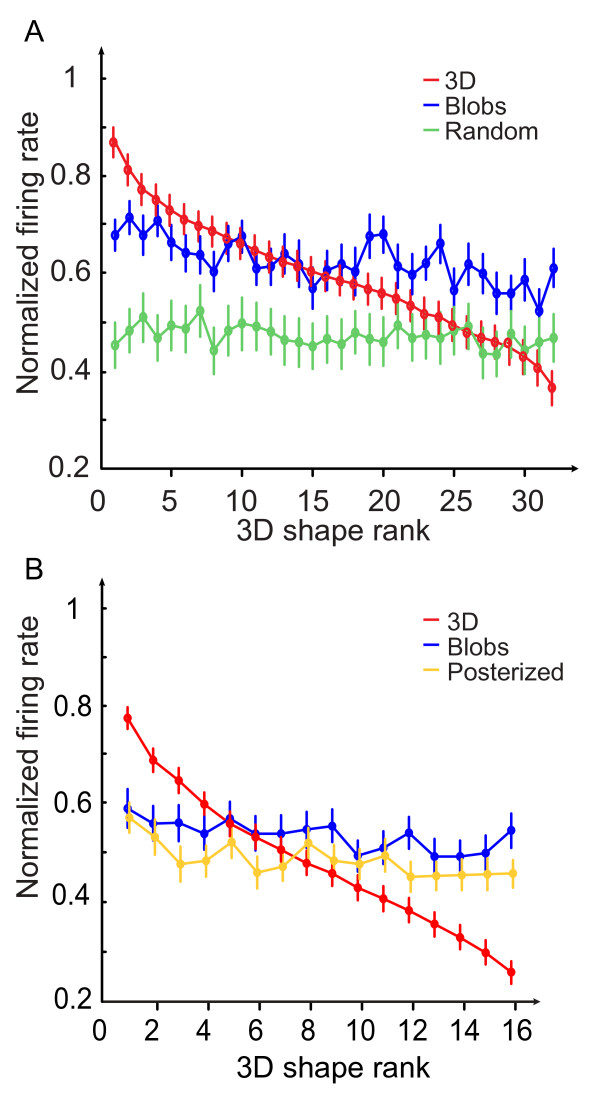
**Rank analysis**. Rank order plot of the 'content' population responses plotted separately for the different classes of stimuli. The red curve illustrates the ranking of 3D stimuli in descending order from average normalized responses (n = 40). Rank 1 corresponds to the preferred 3D stimulus. The same rank is preserved to plot the corresponding responses to the Blob, Random and Posterized stimuli (blue, green and yellow curves respectively). (A) The population of cells tested with the random control stimuli (n = 40). (B) The population of cells tested with the Posterized control stimuli (n = 53).

Considering the results presented so far, it is not obvious that we can draw a firm conclusion about the significance of 3D shape from shading in V4. The comparisons of ω^2 ^indices suggest that the selectivity to shapes is still present for 2D Blob controls and, to a lesser extent, for Random or Posterized stimuli. However, this does not necessarily mean a neuron shows the same shape preference across difference stimulus classes. For example, the neuron in Figure 7A is weakly tuned to 3D shapes (ω^2 ^= 0.06) and more tuned to 2D Blobs shapes (ω^2 ^= 0.12). However, its response is maximal for 3D shape #4 whereas it is maximal for 2D Blobs shape #6. This apparent mismatch between the tuning to 3D and to controls is due to the fact that ω^2 ^is a measure of the magnitude of the selectivity but it makes no assumption on the preservation of the tuning across the different types of stimuli. In order to assess the preservation of the tuning across control stimuli, we performed a rank analysis. To achieve this, we plotted the normalized mean responses of the 'content' cells population as a function of stimulus rank and content of stimuli. For each neuron, we ranked all 3D shapes according to the mean response to its preferred shape in descending order (most effective stimulus was assigned a rank of 1 whereas the least had a rank of 32 or 16, depending on the number of stimuli presented). The resulting stimulus rank order was then applied to the normalized responses to both types of control stimuli (Fig. 8). After ranking the stimuli in all content conditions using 3D shape as a reference for each neuron, the normalized responses were averaged for each rank across all the content selective neurons (n = 93). Figure 8A shows the rank order plots of the stimuli obtained from the normalized responses of the 'content' population tested with Random stimuli (n = 40 cells). As expected, the responses to 3D stimuli on which the rank is based, decrease monotonically as a function of rank. The response curves to Blob and Random stimuli are flat and do not show any significant modulation as a function of stimulus rank (1-way ANOVA, p = 0.152 and p = 0.999 respectively). As 3D shape was the reference for ranking the curves, these results suggest that a given neuron does not have the same order of preference for 3D shapes and corresponding 2D controls. This conclusion is also valid for Posterized stimuli: Figure 8B shows the rank order plot obtained with the 'content' population tested with Posterized stimuli (n = 53 cells). As shown previously in Figure 8A, although the responses to 3D stimuli decrease monotonically as a function of rank, mean responses to Blobs and Posterized stimuli do not vary as a function of rank (1-way ANOVA, p = 0.690 and p = 0.516, respectively). Hence, the rank analysis shows that, whereas the 3D stimuli and the corresponding 2D stimuli (Blobs, Random and Posterized) share a common set of parameters (mean luminance, contrast and layout of black and white patches), the average stimulus preference is different between 3D stimuli and their corresponding 2D controls suggesting that the responses to 3D stimuli could not result only from low level parameters or spatial arrangement of black and white patches.

**Figure 9:**
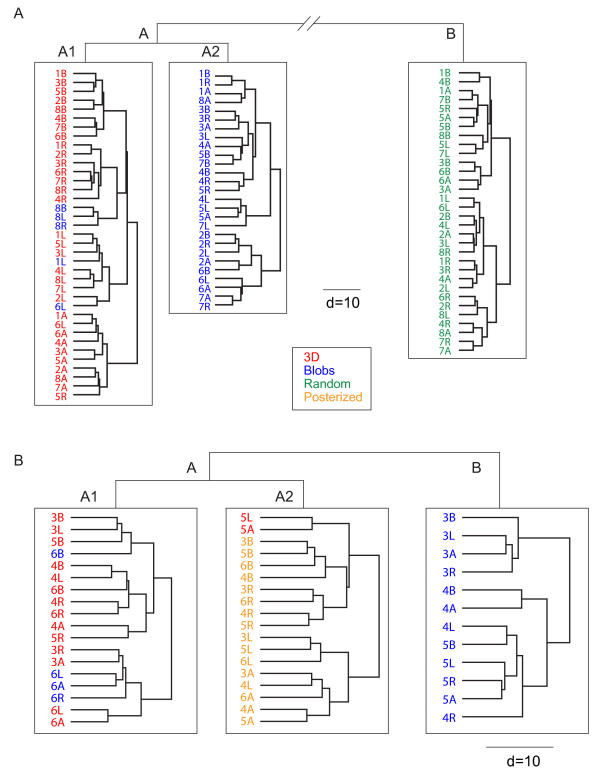
**Hierarchical cluster analysis**. (A) Dendrogram from the hierarchical cluster analysis of the 'content' population tested with Random stimuli (n = 40 cells). Each horizontal branch represents a stimulus (first number) with one direction of illumination (B, L, R or A). Clusters A and B are separated by a linkage distance (d) of 170. At a second level (d = 38), A branch splits into 2 clusters A1 and A2. Each color corresponds to one type of stimuli (3D, Blobs, Random or Posterized). (B) Dendrogram from the hierarchical cluster analysis of the 'content' population tested with Posterized stimuli (n = 53 cells). Same conventions as A.

In order to better visualize the population response, we applied a hierarchical cluster analysis (Ward method, see Methods) on the standardized responses of the 40 'content' cells tested with random controls (96 stimuli: 32 3D + 32 Blob + 32 Random). Figure 9A shows the dendrogram in which each terminal branch of the tree represents one stimulus. A short linkage distance (d) between two stimuli means that the V4 neuronal population treats these stimuli as very similar. Globally, the tree splits into two distinct clusters (A and B) at its highest level (d = 170). The A branch contains all the 3D (red) and Blob (blue) stimuli while the whole set of 2D Random (green) stimuli belongs to cluster B. At the next level (d = 38), the A branch splits into two distinct clusters A1 and A2 gathering 37 and 27 stimuli respectively. A1 contains all 3D stimuli and only 5 2D Blob stimuli whereas A2 contains only 2D Blob stimuli. Finally, and most interestingly, A1 is subdivided into 4 branches that contain 3D stimuli with identical lighting directions (B, R, L, A clusters in descending order). Only two 3D stimuli are misclassified, stimuli 6L and 5R. Notably, the respective lighting directions of control stimuli are not clustered.

This analysis gives strong indications that 3D and 2D stimuli are well separated by the responses within the V4 population. This separation cannot be explained in terms of mean luminance and power spectrum differences between 3D and Blob stimuli. Nevertheless one could claim that the spatial distribution of grey levels is a determinant factor in the differential clustering of 3D vs. 2D because of the known sensitivity of V4 neurons to the phase of visual stimuli [31]. Indeed, although 3D stimuli are easily distinguishable from 2D Blob stimuli by a vivid 3D aspect because of the shading, the spatial distribution of dark and light patches is very different in both types of stimuli. This is the reason why we designed the Posterized control stimuli that respect more the polarity of the 3D stimuli. Figure 9B shows the result of the hierarchical cluster analysis performed on the 53 'content' cells tested with Posterized stimuli. As for the subpopulation of cells displayed in Figure 9A, most stimuli have a strong tendency to be clustered by V4 cells according to their type. The tree splits at the first level (d = 43) in two distinct clusters (A and B), where A contains all 3D and Posterized stimuli and B contains exclusively Blobs stimuli (12 out of 16). At a lower level (d = 29), cluster A splits in 2 subgroups A1 and A2, each containing 18 stimuli. A1 is composed of all but two 3D stimuli (14/16) in addition to Blob controls of shape #6 whereas all Posterized stimuli are found in cluster A2. Interestingly, directions of illuminations have a marked tendency to be grouped in this cluster of Posterized stimuli. Considering the distances between clusters, we found that 3D shapes are closer to Posterized stimuli than to 2D Blob stimuli whereas in both trees the distance between 3D shapes and Blob stimuli are similar (38 and 43 for Figure 9A and 9B, respectively). Responses to Random stimuli are markedly different from responses to other types of controls with a cluster separated from the 3D cluster by a long distance of 170.

## Discussion

The main result of our study is that 3D stimuli defined by shape from shading are distinct from 2D controls by population coding in V4. This reflects the importance of this mid-level area of the "object information processing pathway" in the elaboration of this complex visual attribute.

First, our results show that single cell selectivity to the 3D shapes used in this study is broad as determined by the SSI and ω^2 ^indices. Although a vast majority of the cells are efficiently driven by the pool of stimuli, only 45% are statistically modulated by 3D shapes and even fewer can individualize a given 3D shape (23 neurons according to the ω^2 ^criterion). One possibility of the rather weak occurrence of tuned units is that some parts (similarly oriented curved ridges or prominent bumps) are common to different stimuli, albeit placed in different positions.

Next, whereas the direction of illumination modulates 55% of the cells responses, the selectivity indices (ISI and ω^2^) also show that few individual cells are selective to the direction of illumination. One possible reason of the relatively sparse occurrence of such selectivity is that the complex pattern of shading varies a lot from shape to shape for a given direction of illumination. Another interesting result is that the distribution of Direction indices does not reveal any preference for a given direction of illumination. Hanazawa and Komatsu (2001) demonstrated that a majority of V4 neurons exhibited sensitivity to the direction of luminance gradients in 3D texture patterns that was biased towards the vertical gradient [17]. We suggest that, because our stimuli contained several complex curves, the source of illumination may not be as obvious as it would be with Hanazawa's textures.

Since most individual cells are broadly tuned to illumination direction, one could expect they achieve invariance to illumination. Our results show that the few cells that are strongly shape selective according to the ω^2 ^criterion (> 0.10) have a tendency to be invariant to illumination direction (ie. there was a negative correlation between ω^2 ^indices). The invariance of neuronal discharge according to the different illumination directions is a crucial step in the shape from shading process. Indeed, humans have remarkable abilities to achieve object recognition under different illumination directions and one can assume that macaque monkeys have a similar visual skill. For example, lesion work in the macaque monkey indicated that the inferior temporal cortex is critical for object recognition under varying conditions of illumination [32]. However, the question of invariance in terms of illumination direction is controversial in the literature. The structural theory of recognition suggests that the visual system extracts illumination-invariant features from the scene [33,34]. Psychophysical results are consistent with this theory as humans can recognize objects and, in some cases, faces effortlessly when the direction of illumination varies [9]. On the other hand, image-based theory proposes that direction of illumination is encoded in internal face and object representations [35,36]. This theory is supported by psychophysical data showing that recognition of faces and objects varies with illumination [10,37,38]. The results from the individual cells could well support either theory as we reported the presence of few individual cells that were invariant to direction of illumination but selective to 3D. However, the population analysis did not reflect a counterpart of the 'structural theory': we observed no clustering of individual 3D shapes in the dendrograms obtained from the population responses, suggesting that more computational steps beyond V4 are required to individualize 3D objects lit under different directions. Nevertheless, the results of the cluster analysis showed a tendency for the same illuminations to be grouped together at the last branches of the dendrogram for 3D stimuli in Figure 9A, suggesting a tendency to code the direction of luminance in complex shapes. However, we note that, in Figure 9B, the effect is not present for 3D stimuli but for Posterized stimuli only. Although one cannot rule out a sampling bias issue, this may also reflect the fact that the polarity of dark and light regions is more obvious in Posterized than in the 3D shape stimuli. Hence, the cluster analysis may be revealing the mechanism that underlies the extraction of illumination direction in complex shading patterns. Such mechanisms would fit predictions of image-based theory. However, our results are limited in the sense that the monkeys performed a passive fixation task. It would be an interesting development of this study to demonstrate that invariance to a broader range of angles of illumination can be obtained in an active recognition task. To accomplish this, an experiment would have to be designed in which monkeys would be trained to recognize individual objects (of the kind we used) under various illuminations. This generalization of object recognition to 'difficult' illumination is plausible in V4 since neurons of this area have been demonstrated to be prone to perceptual learning [39].

At this stage, it is difficult to argue in favor of real 3D coding in V4. The 3D rendering of our stimuli is very vivid because of the strong shading gradients. Thus, illumination direction and 3D shape are strongly linked by construction of the stimuli and, as such, they are unavoidably intermixed. The controls we used for the 3D shape from shading stimulus were created by disorganizing the structure of the image while trying to keep the same low-level parameters. Whenever a neuron (or a population of neurons) is selective to the 3D stimuli and not to (or separated from) Posterized and Blob stimuli, it means that the gradient of tones alone or the pattern of dark and light patches alone are not sufficient to drive the cell. This would suggest that this cell could be an important step of processing shape from shading. Our results show that a vast majority (78%) of V4 neurons responded differently to 3D stimuli and these 2D control versions. However, the ANOVA and the Tukey test show that there is a comparable number of cells that prefer 2D controls (Blobs and Posterized) as prefer the 3D stimuli. This point needs to be emphasized in regards to the results of Georgieva and colleagues in fMRI [13]. In humans, many regions sensitive to 3D shapes were also responsive to 2D shapes, and this was likely the case in the area equivalent to V4 of the macaque monkey. If the respective global responses of two separate but intermixed neuronal populations (in the present case our 3D- and 2D-biased neurons) have the same strength, the resulting pattern in fMRI will not be able to identify a 3D selective region [40]. We recorded a subset of only 24 neurons that displayed a clear individual preference for 3D stimuli. The presence of this subpopulation is consistent with the results of Georgieva and colleagues who report that activation related to shape from shading can be found in ventral areas [13], although, besides the quite complex problem of homologies between species [41], the main focus of activity is likely to correspond to a more anterior region in the macaque.

When responses of V4 cells are analyzed at the population level, we obtain better evidence that neurons differentially encode 2D stimuli and 3D stimuli defined by their shading. This is shown firstly in the rank analysis where responses to 2D stimuli were ranked according to the 3D stimuli preference. We first observed that the ranking of 3D stimuli is clearly different from that of 2D Blobs and Random stimuli. This rules out the possibility that the selectivity to 3D shapes is based only on low level parameters and suggests that the disposition of the dark and light regions, very different in each type of stimuli, is important for V4 cells. We then observed that the ranking of 3D stimuli does not match that of Posterized stimuli either. This suggests that the gradient of grey levels, absent in the two-tones Posterized stimuli, is also important. A better visualization of the respective coding of each stimulus type is provided by the cluster analysis. This suggests that the V4 population is able to accurately discriminate between the different types of stimuli. The 3D stimuli and the different classes of 2D controls mostly belonged to different clusters, suggesting that the population response gives a separate status to the 3D stimuli. The different clusters cannot be explained by low-level parameters only; although 2D Blob stimuli had the same first order parameters as the 3D stimuli (this may explain the correlation between the rank plots), we did observe two clusters corresponding to each class of stimulus. Similarly, Random stimuli, which have the same mean luminance and contrast but differ markedly from 3D and Blobs in their spatial frequencies, belong to a cluster that was separated from the other two by a distance four times longer than that between the 3D shapes and blob stimuli. The question remains: Why are the 3D stimuli separated? Although each 3D stimulus is easily recognizable from the others as they are defined by a different inner content, all stimuli also could be considered as being covered by the same texture or material (here a kind of glossy metal). Our recent work [24] has stressed the fact that V4 cells can classify natural textures, and others have shown the significance of the human equivalent for attention to surface properties [42]. If the 3D stimuli were treated by V4 as a texture, we should expect them all to have the 'special status' revealed by the cluster analysis. But we think that our results show more than a mere coding of a particular texture. The cluster analysis shows that the Posterized stimuli are closer to 3D stimuli than 3D are to Blob stimuli. This suggests that the polarity of the dark and bright patterns on the stimuli (similar in 3D and Posterized only) matters more than low-level parameters in the classification.

Hence, both rank and cluster analysis point to the significance of the disposition of dark and light patches together with a gradient of grey levels. This double selectivity is an important stage to perceive shape from shading as a given direction of illumination on an irregular surface results in a unique shading pattern. However, the question remains open as to whether our results reflect a genuine coding of 3D shape (from shading) per se. Many groups have shown the importance of depth encoding in V4. For instance, V4 cells are selective to disparity or 3D orientation of bars [43,44]. However individual V4 cells may not explicitly represent orientation of curvature in depth when depth is coming from disparity [45]. In our study, the monocular depth cue was brought about by illumination and similarly, and the V4 cells could not achieve complete shape invariance. Using stimuli similar to ours while recording in TEO, Vangeneugden and colleagues [46] did not observe striking differences to our results except that a majority of cells prefer 3D-shapes over the controls. However a previous study reports depth-invariant shape selectivity in area the infero-temporal cortex [47]. It may be that the complex percept of 3D shape from shading needs to build up through V4 and TEO stages before reaching invariance in IT. In this case, area V4 could encode 3D cues like shading, texture gradient or disparity and send this information to infero-temporal cortex [48,49]. However, it is not yet completely understood how shape and surface selectivities build up through early levels, V4 (as a putative intermediate stage) and different IT subregions [50-52]. One very important point that remains unclear is what areas contribute to the vivid naturalness of the phenomenological percept of 3D. This remains to be tested with behavioural tasks [23] while focusing in the regions corresponding to human posterior LOC, which is a region of high convergence of 3D cues [13].

## Conclusion

This study shows that area V4 of the monkey plays a significant role in the cortical processing steps leading to perception of 3D objects defined by shape from shading. The shape from shading selectivity that is not obvious at the level of the single cell is suggested at the population level.

## Authors' contributions

FA and CJ carried out recordings. FA, CJ and PG participated in the design and technical setup of the study, including animal surgery and training. FA, CJ and PG performed data analysis and participated in writing the manuscript. All authors read and approved the final manuscript.

## Supplementary Material

Additional file 1 **Table S1**. Mean population response ± SD to stimuli types (3D or controls) for each cell type (left column) determined by the ANOVA. Click here for file
